# LATE IMPACT OF THE LAPAROSCOPIC TREATMENT OF DEEP INFILTRATING ENDOMETRIOSIS WITH SEGMENTAL COLORECTAL RESECTION

**DOI:** 10.1590/0102-672020180001e1406

**Published:** 2018-12-06

**Authors:** Antonio Matos ROCHA, Maurício Mendes de ALBUQUERQUE, Eduardo Miguel SCHMIDT, Cristiano Denoni FREITAS, João Paulo FARIAS, Fernanda BEDIN

**Affiliations:** 1 Medical School, University of Southern Santa Catarina, Palhoça;; 2 Surgery Service, Bahia Sul Hospital, Florianópolis, SC, Brazil

**Keywords:** Endometriosis, Colorectal surgery, Laparoscopy, Endometriose, Cirurgia colorretal, Laparoscopia

## Abstract

**Background:**

Deep infiltrating colorectal endometriosis may severely affect the quality of life and fertility of patients. Although segmental resection is a therapeutic option that provides positive outcomes in the management of symptoms, its functional effects are still unproven.

**Aim:**

Assess the late impact of the laparoscopic approach in treating deep infiltrating endometriosis with segmental colorectal resection.

**Methods:**

Prospective case series of 46 patients submitted to laparoscopic treatment of deep infiltrating endometriosis with segmental colorectal resection between 2013 and 2016. Fertility, gynecological and bowel symptoms were assessed at the preoperative period and at three and 12 months (or more) after the procedure.

**Results:**

Preoperative interview assessed the prevalence of infertility (45.6%), gynecological (87%) and intestinal (80.4%) symptoms. At the third month after the procedure a significant reduction in the prevalence of gynecological symptoms (p<0,001), tenesmus (p=0,001) and dysquesia (p=0,002) was observed. After a period of 12 months or more following the procedure a significant reduction in the prevalence persisted for dysmenorrhea (p=0,001), deep dyspareunia (p=0,041), chronic pelvic pain (p=0,011) and dysquesia (p=0,001), as compared to the preoperative period. Total pregnancy rate was 57.1% and spontaneous pregnancy 47.6%.

**Conclusion:**

The treatment of deep infiltrating endometriosis using segmental colorectal resection has provided early and late relief of gynecological and bowel symptoms. The outcomes also indicate a positive impact on the fertility of infertile patients.

## RESUMO

**Racional:**

A endometriose profunda infiltrativa colorretal pode impactar de maneira importante na qualidade de vida e na fertilidade das pacientes. A ressecção segmentar é uma opção terapêutica com resultados positivos na queda dos sintomas, porém ainda sem efeitos funcionais comprovados.

**Objetivo:**

Avaliar o impacto tardio do tratamento laparoscópico da endometriose profunda infiltrativa com ressecção segmentar colorretal.

**Métodos:**

Série de casos prospectiva com 46 pacientes submetidas ao tratamento laparoscópico para endometriose profunda infiltrativa com ressecção segmentar colorretal entre 2013 e 2016. Foram analisados sintomas ginecológicos, intestinais e a fertilidade no período pré-operatório, três e 12 meses ou mais após o procedimento.

**Resultados:**

Na entrevista pré-operatória, foram levantadas as prevalências de sintomas ginecológicos (87%), intestinais (80,4%) e de infertilidade (45,6%). No 3º mês pós-operatório, observou-se redução significativa da prevalência dos sintomas ginecológicos (p<0,001) e de sintomas intestinais, tenesmo (p=0,001) e disquesia (p=0,002). Após 12 meses ou mais observou-se diminuição significativa da prevalência de dismenorreia (p=0,001), de dispareunia profunda (p=0,041) e de dor pélvica crônica (p=0,011) além de disquesia (p=0,001) em relação ao período pré-operatório. As taxas de gravidez total e espontânea foram de 57,1% e 47,6%, respectivamente.

**Conclusão:**

O tratamento da endometriose profunda infiltrativa com ressecção segmentar colorretal proporcionou alívio precoce e tardio dos sintomas ginecológicos e intestinais. Os resultados sugerem impacto positivo sobre a fertilidade em pacientes inférteis.

## INTRODUCTION

Deep infiltrating endometriosis (DIE) is defined as the presence of endometrial glands and stroma outside the uterine cavity penetrating adjacent structures to a depth of 5 mm or more^15^. Its prevalence is growing and, nowadays, it is estimated that 10-15% of women of reproductive age are affected by the condition^14^. The infiltrating form of the disease affects around 20% of women with endometriosis^5^.

Colorectal involvement due to DIE represents one of the most severe forms of the disease and, when present, is often associated with other DIE lesions in the pelvic cavity^14^.

Clinically, it presents itself through gynecological symptoms such as dysmenorrhea, chronic pelvic pain, and deep dyspareunia, which may or may not be associated with bowel symptoms such as dyschezia, cyclic hematochezia, constipation, diarrhea, and tenesmus. It may inflict a severe impairment in the quality of life, apart from being one of the chief culprits of infertility^10^.

The treatment for colorectal form should be individualized according to the severity of symptoms, location, extent and depth of lesions, as well as taking into consideration the patient’s expectations^3^. In most of the cases, initial clinical treatment should be considered with the aim of managing symptoms, reducing lesions and/or controlling the progression of the condition^2^. Clinical treatment alone, however, is not able to eradicate lesions and requires chronic and prolonged use of medication, which in turn can cause side-effect and have a significant impact on the reproductive capacity of patients^2^ ^,^ ^11^ ^,^ ^22^.

Failure in the clinical treatment, the wish of infertile patients to get pregnant, the presence of lesions with transmural invasion with risk of obstruction, and digestive bleeding are some of the indications for surgical treatment^3^. 

Among surgical options, laparoscopic segmental resections, shaving and disc resections have been used and favored by different authors^4^ ^,^ ^25^. Considering the benign nature of endometriosis, some groups advocate for more economical options, such as shaving or disc resections, with the aim of preserving both anatomy and function, notwithstanding that relapse-prone endometrial tissue may remain on the operative site^7^ ^,^ ^25^.

Growing evidence demonstrate that the best outcomes related to symptoms management and quality of life improvement are reached with the complete excision of all identified endometrial implants, achieved through a combined approach between surgeons and gynecologists^1^ ^,^ ^20^.

Minor and shallower lesions are eligible to less aggressive colorectal resections, but in the case of more extensive and circumferential or multifocal involvement, these techniques may not be an option and segmental resection may be the only possible alternative^4^. Conversely, segmental resections may be responsible for the appearance of new colorectal symptoms and complications, and possibly higher postoperative morbidity^27^.

Whereas some authors insist in the wide use of shaving and disc resection, others sustain that segmental resection, for moderate and severe cases, present comparable symptomatic outcomes^20^.

This paper aims at evaluating the late impact of laparoscopic segmental colorectal resection in the treatment of DIE.

## METHODS

The study was approved by the ethics committee of the Universidade do Sul de Santa Catarina, under registration 1.634.466.

This study is based on a prospective case series of DIE patients submitted to laparoscopic segmental colorectal resection carried out at Florianópolis, SC, Brazil, between January 2013 and April 2016. All patients diagnosed with DIE with colorectal involvement as established by imaging tests (transvaginal ultrasound with bowel preparation and/or pelvic MRI) and whose laparoscopic findings were compatible and confirmed by anatomopathological analysis, were included in the study.

Patients were followed and questioned about specific gynecological and intestinal symptoms prior to the operation, at the third postoperative month, and by the last medical consultation, 12 or more months following the surgery. Information was also gathered concerning parity, whether an infertility diagnosis had been made, and on pregnancies after the surgery.

All patients were operated by a team of gynecologists and digestive system surgeons and had all visible endometrial lesions resected. Gynecological intervention involved the resection of ovary endometriomas and other pelvic lesions and, in selected cases, chromotubation, myomectomy or hysterectomy. All segmental colorectal resections were carried out by the same surgeon. Ureter and hypogastric nerves were systematically identified and preserved, except when directly compromised. Distal margins were defined right under the last endometrial rectal lesion. When present, peritoneal lesions in the posterior pelvic cavity, including implants in the rectovaginal septum and uterosacral ligaments, were resected along with the surgical specimen, which were exteriorized through Pfannenstiel incisions. In all cases mechanical circular anastomosis were performed. Ileostomy was opted for whenever anastomoses were 5 cm or less from the anal margin. All anatomopathological exams confirmed the disease and the presence of free margins.

 Patients not intending to get pregnant received postoperative hormone blocking therapy. Those wishing to get pregnant sought natural or in vitro fertilization.

### Statistical analysis 

For the descriptive analysis, qualitative and quantitative variables were described through absolute and relative frequencies. Differences in the prevalence of gynecological and bowel symptoms at preoperative and postoperative stages were compared. McNemar’s chi-square test was used to evaluate the associations among variables and the differences among proportions (pre and post). A 5% (p<0.05) confidence level was determined.

## RESULTS

Forty-six patients diagnosed with DIE with colorectal involvement whose laparoscopic findings were compatible and confirmed by anatomopathological examination were included in the study. All colorectal specimen presented disease free margins. Mean postoperative follow-up was 28.4 months.

Mean age was 34.28 years, varying from 19 to 45. In regards to parity, 33 were nulliparous (71.7%), seven primiparous (15.2%), and six multiparous (13%). In the preoperative interview, 87% of patients reported gynecological symptoms, 80.4% bowel symptoms and 45.6% infertility.

Early postoperative complications occurred in five patients (10.8%), of which three were colorectal anastomotic fistulas, one bleeding and one bowel obstruction. In four of these cases, laparoscopic intervention was needed. Loop ileostomy was performed on six patients, three of which due to anastomotic fistula and the other three due to the proximity of the colorectal anastomosis to the anal verge (5 cm).

Table 1 shows the prevalence of symptoms in the preoperative period and after three months of the surgical procedure. A significant reduction in the prevalence of all gynecological symptoms was observed (p<0.001). Likewise, dyschezia and tenesmus, the most common bowel symptoms, showed a significant prevalence reduction (p=0.001 and p=0.002, respectively). Among patients presenting preoperative cyclic hematochezia, none reported it at the third postoperative month.

**TABLE 1:** Symptoms at pre- and three months postoperative

Symptom	Pre-op n(%)	3rd month post-op n(%)	p
Gynecological			
Dysmenorrhea	25 (54.3)	03 (6.5)	<0.001
Dyspareunia	22 (47.8)	03 (6.5)	<0.001
Chronic pelvic pain	21 (45.7)	02 (4.3)	<0.001
Bowel			
Dyschezia	17 (37.0)	03 (6.5)	0.001
Cyclic hematochezia	09 (19.6)	0 (0)	
Constipation	11 (23.9)	02 (4.3)	0.012
Diarrhea	05 (10.9)	02 (4.3)	0.453
Tenesmus	23 (50.0)	07 (15.2)	0.002

The prevalence of symptoms 12 months or more after the surgical procedure in comparison to the preoperative period are shown in Table 2. Dysmenorrhea, deep dyspareunia, and chronic pelvic reduced significantly (p<0.05). When the most reported intestinal symptoms are analyzed, a significant reduction is observed for dyschezia compared to the preoperative period (p=0.019). No patient reported cyclic hematochezia. The prevalence of constipation, diarrhea, and tenesmus did not present significant differences.

**TABLE 2:** Symptoms at pre- and at 12 or more months post-operative

Symptom	Pre-op n(%)	≥12 months PO n(%)	p
Gynecological			
Dysmenorrhea	25 (54.3)	10 (21.7)	0.001
Dyspareunia	22 (47.8)	12 (26.1)	0.041
Chronic pelvic pain	21 (45.7)	08 (17.4)	0.011
Bowel			
Dyschezia	17 (37.0)	06 (13)	0.019
Cyclic hematochezia	09 (19.6)	0 (0)	
Constipation	11 (23.9)	12 (26.1)	1
Diarrhea	05 (10.9)	03 (6.5)	0.625
Tenesmus	23 (50.0)	19 (41.2)	0.481

By grouping gynecological and bowel symptoms (Table 3) there was an increase in the prevalence of all symptoms when the third postoperative month is compared to the period of 12 or more months after the surgery (p<0.05). When analyzing the prevalence of symptoms in the comparison between the preoperative and the latest period (Table 4), it is observed that both gynecological and bowel ones show significant statistical differences (p<0.001).

**TABLE 3:** Symptoms at three and at 12 or more months post-operative

Symptom	3^rd^ month n(%)	≥12 months PO n(%)	p
Gynecological			
Yes	6 (13)	20 (43.5)	0.003
No	40 (87)	26 (56.5)	
Bowel			
Yes	11 (23.9)	23 (50)	0.023
No	35 (76.1)	23 (50)	

**TABLE 4:** Symptoms at pre- and at 12 or more months post-operative

Symptom	Pre-op n(%)	≥12 months PO n(%)	p
Gynecological			
Yes	40 (87)	20 (43.5)	<0.001
No	6 (13)	26 (56.5)	
Bowel			
Yes	37 (80.4)	23 (50)	0.001
No	9 (19.6)	23 (50)	

Fertility data is shown in Table 5. It can be seen that from the 33 nulliparous, 19 were infertile and yet, 57.5% of these infertile women got pregnant 12 or more months after surgical treatment. Total pregnancy rate was 57.1% and spontaneous pregnancy 47.6%. Two patients reported getting pregnant following in vitro fertilization. 

**TABLE 5:** Fertility at pre- and post-operative

Parity (n)	Pregnancy in post-op n(%)	Pre-op infertility n(%)	Post-op pregnancy among pre-op infertile n(%)
Nulliparous (33)	14 (42.4)	19 (57.5)	11 (57.9)
Primiparous (7)	1 (14.3)	2 (28.5)	1 (50)
Multiparous (6)	0 (0)	0 (0)	0 (0)

## DISCUSSION

Endometriosis affects predominantly young women of fertile and economically active age and causes severe symptoms which can potentially limit daily activities. The disease has a markedly negative impact on the quality of life and affects several aspects of daily life including sexuality and work performance^10^. When evaluating patients submitted to segmental colorectal resection to treat DIE, studies can detect a significant improvement in the quality of life and positive impacts in sexuality, family dynamics, and work performance^13^ ^,^ ^16^ ^,^ ^23^. National studies also demonstrate improvement in quality of life scores up to 48 months after segmental resection compared to preoperative scores^23^ ^,^ ^26^.

The present study used the prevalence of symptoms as reported at preoperative stage and at three and 12 (or more) months intervals following surgical procedure as a proxy for evaluating the impact of segmental colorectal resection. In the short-term, an important and significant reduction in the gynecological and bowel symptoms previously identified in the preoperative stage was observed. Similarly, other studies have noticed a significant reduction in gynecological (dyspareunia and dysmenorrhea) and intestinal (dyschezia) symptoms three months after the procedure^4^ ^,^ ^13^. 

When symptoms reported 12 or more months following the resection are compared to the preoperative reports, a marked drop in the prevalence of dysmenorrhea, dyspareunia, chronic pelvic pain, and dyschezia could be observed. A special note has to be made to cyclic hematochezia, a symptom reported by around 20% of patients prior to the surgery and by none after it, as a strong indicator to the effectiveness of the procedure in the management of the clinical manifestations directly related to colorectal DIE. Three other studies, with average follow-up periods between 12 and 24 months, evidenced similar findings^4^ ^,^ ^13^ ^,^ ^16^.

However, in the period between three and 12 or more months after the colorectal resection, an increase in the prevalence of gynecological and bowel symptoms was observed, more specifically constipation and tenesmus, with rates similar to those found at the preoperative period. Along the same line, Kent *et al* ^13^ *.* noticed a trend in the relapse of symptoms 12 months after the surgery, a period at which Kössi *et al.* ^16^ identified a 27% prevalence of constipation^,^. Another relevant study found a significant worsening of constipation symptoms in patients who underwent segmental resections compared to patients in a control group^27^.

These findings may lead to inferring that these symptoms may stem from a different cause, other than endometriosis, or even that they bear no causal relation to the operation performed. The same reasoning may apply to tenesmus, which also fail to present statistical difference in the later period. Other variables, such as dietary and behavioral habits, tend to be associated; postoperative recommendations, meant to optimize bowel function, have a tendency to be followed more attentively in the initial phases of the postoperative period before patients relapse back into preoperative old habits later on. There is also evidence that irritable bowel syndrome is more frequent in women with endometriosis^30^.

A study by Soto *et al.* ^27^ observed dysmenorrhea and chronic pelvic pain in about 30% of cases four years after colorectal resection without, however, having any statistical difference compared to controls. This study also identified a significant worsening in constipation when patients were directly asked about the presence of this symptom, which could not be proved after a detailed assessment was carried out using validated and standardized qualitative questionnaires.

Other published papers have also demonstrated that a portion of patients still had complaints about dyspareunia, dysmenorrhea, chronic pelvic pain, and dyschezia in the mid-term^4^ ^,^ ^8^ ^,^ ^16^. In spite of this, the majority of patients who still had the manifestations reported a reduction in the intensity of the symptoms^8^ ^,^ ^16^. The present study was limited to establishing the presence or absence of symptoms and, since no validated standardized quantitative questionnaire was used, represents an estimation of the surgical intervention’s impact on the symptoms without, however, attempting to quantify them.

Painful complaints may be associated to a relapse of the disease. One study identified a reoperation rate of 19.4% on patients previously operated^8^. Another demonstrated that laparoscopically proven relapse affected 6.6% of patients who had undergone segmental resection within an average period of 24 months. All re-intervention referrals were prompted by pain^4^. Young patients with high BMI and positive surgical margins seem more prove to relapse^21^.

The significant management of painful symptoms up to the third month demonstrated the impact of the surgical intervention. In the late postoperative (mean of 28.4 months), there is the possibility that the recurrence of the disease affected mainly patients who, wishing to get pregnant, did not receive hormone blocking therapy, but also failed to get pregnant. During this time, there was an opportunity for the resurgence of new implants or reactivation/growth of a microscopic residual source. A study by Malzoni *et al.* ^18^ demonstrates a higher pelvic relapse rate in patients without postoperative hormone suppression. In the present study, dyspareunia stands out as the most relevant symptom, recurring to 26% of patients, though there was a statistically significance in relation to the preoperative.

At present, with the refinement of surgical techniques and the increasing body of scientific evidence, it is a consensus that the invasive treatment of DIE requires a complete resection of the disease^1^ ^,^ ^2^ ^,^ ^13^, preferably with less aggressive surgeries that can preserve to a maximum both anatomy and function^7^ and serve as an important adjuvant in the treatment of infertility^6^. According to Roman *et al.* ^24^, the treatment of colorectal endometriosis should be based on the reduction of pain, rather than on its replacement by other symptoms.

The surgical indication should be determined individually and should be based on the severity of the painful manifestation of symptoms, the extent of the disease as established by the findings of transvaginal ultrasound with bowel preparation and/or MRI, and the expectations of the patient in regard to pregnancy and symptoms’ managment^17^. The definite line of actions should be guided by the intraoperative findings^29^.

Robust evidence have being published reinforcing the case for shaving and disc resection given the remarkable outcomes in regards to symptom management and fertility^7^ ^,^ ^9^ ^,^ ^25^. The option for less aggressive approaches prevents mesocolon and mesorectum incision, active organ mobilization and the potential injury to pelvic nerve structures, which may lead to sexual, urinary and digestive function, with relevant morbidity^25^. In this context, segmental resection represents potential risk and occupies an increasingly more defined space.

Another important variable that may affect outcome is the height of the rectal resection. A review of the literature has shown that bowel dysfunctions occur more frequently in patients submitted to lower resections, with the worst long-term functional outcomes associated to rectum approaches compared to those performed at the sigmoid level^12^ ^,^ ^22^. This suggest that the modification of the reservoir capacity of the rectum may bring additional functional impairments, other than those occasionally caused by nerve damage. The present study, however, did not aim at the detailed analysis of this variable.

However, a comparative study of the three main laparoscopic techniques, though finding that relapses were associated to all of them, showed that they are less severe when resections are segmental, which may be due to the incomplete excisions by shaving or disc resection^4^. Malzoni *et al.* ^18^ *,* in a series of 248 patients, demonstrated that segmental colorectal resections were effective in reducing pain and restoring bowel function. A study by Meuleman *et al.* ^20^ also demonstrated low rates for complications and relapse/reintervention and good fertility outcomes associated with segmental resections in moderate and severe cases. Similarly, Roman *et al.* ^24^ assert that conservative approaches should not prevail in all cases, since larger and more complex lesions should be preferably submitted to segmental resections due to the high relapse risk.

A recent publication has shown that the preservation of the hypogastric plexus and the mesorectum is capable of maintaining bowel, urinary and sexual functions, reducing morbidity, maintaining cure rates and improving the quality of life^19^. Procedures of such technical refinement require experienced and well trained professionals^18^ ^,^ ^29^.

In the present study, all resections involved multiple lesions, deep or circumferential, thus with precise indication for segmental resection. Furthermore, all studied population was comprised of patients diagnosed beforehand with colorectal involvement and, therefore, excluded patients submitted to exclusively gynecological laparoscopy within which occasional endometrial lesions could have been found in the colon or rectum. In these cases, minor and less complex lesions could be addressed with shaving or disc resections.

Another cautious observation to be made is that, associated with segmental colorectal resections, other equally important interventions were performed on concomitant pelvic lesions, such as the excision of ovarian endometriomas and the resection of lesions of the uterosacral ligaments and the rectovaginal septum. Such interventions should also be considered as responsible for much of the impact on the management of symptoms, especially painful ones.

In addition to the painful and functional symptoms, infertility is an important complaint among patients with endometriosis. In the context of bowel involvement, authors suggest that colorectal lesions have a negative impact on fertility, which can be attributed, at least in part, to the obliteration of the rectouterine pouch^28^.

The present study has found that 45.6% of patients presented with infertility complaints, of which 90% were primary. Total pregnancy rate was 57.1% and spontaneous pregnancy 47.6%. A systematic review conducted to assess the impact of colorectal surgery on the fertility of patients with DIE, has found a spontaneous pregnancy rate of 40-60%. According to the authors, despite the absence of randomized studies, the positive impact may not be ruled out^11^.

A study conducted by Stepniewska *et al.* ^28^ noticed a significantly higher pregnancy rate among patients with bowel involvement submitted to resection (35%) compared to those who were not submitted to digestive intervention (21%), and higher still in patients without bowel involvement (70%). These outcomes suggest that the total excision of intestinal lesions brings benefits in regards to fertility.

In addition to a positive influence on the rate of spontaneous pregnancy, authors suggest that the surgical treatment also increases the success rate of artificial reproduction techniques^11^ ^,^ ^28^. In the present study, only two infertile patients reported pregnancy after in vitro fertilization. As the option for in vitro fertilization involves considerable economic investment, the pregnancy rate may be influenced by this factor, as well as others not related to endometriosis. Darai *et al.* ^6^ state that, in spite of demonstrated positive impact on the improvement of the quality of life and on symptom management, radical surgical treatment should be indicated cautiously, mainly for asymptomatic patients when the sole purpose is to treat infertility, given the likeliness of serious complications.

 As far as postoperative complications are concerned, authors consider that the radical technique poses a low rate of complications that varies from 7.3% to 12%^13^ ^,^ ^18^ ^,^ ^20^ ^,^ ^29^. In the present study, only four patients (8.7%) experienced postoperative complications requiring reoperation, three of which were due to anastomotic fistula and one to incisional hernia. Studies correlate the rate of complications to the extent of the disease^29^. Although considering it a safe procedure, authors state that when considering resection, patients should be properly informed about the possibilities of postoperative complications^29^.

Endometriosis, particularly its infiltrating colorectal form, has a serious impact on the life of patients. This paper, in accordance with other studies, suggests an impact on gynecological and intestinal symptoms of DIE patients with colorectal involvement. Surgical treatment with total resection of all implants promotes clinical improvement and seems to have a positive impact on fertility. Doubts arise about the degree of aggressiveness of this treatment. Scant evidence makes it difficult to standardize the surgical approach, be it conservative or radical. The therapeutic plan should be individualized and guided by clinical and laparoscopic findings and, above all, based on the future expectations of patients in regards to symptom management, fertility and potential impacts of available procedures.

## CONCLUSION

The treatment of DIE with segmental colorectal resection has provided early and late relief of gynecological and bowel symptoms. The results show a positive impact on the fertility of infertile patients.

## References

[1] Abrão MS (2016). Pillars for Surgical Treatment of Bowel Endometriosis. J Minim Invasive Gynecol.

[2] Abrão MS, Borrelli GM, Kho RM, Ceccaroni M, Clarizia R (2017). Strategies for Management of Colorectal Endometriosis. Semin Reprod Med.

[3] Abrão MS, Petraglia F, Falcone T, Keckstein J, Osuga Y, Chapron C (2015). Deep endometriosis infiltrating the recto-sigmoid Critical factors to consider before management. Hum Reprod Updat.

[4] Afors K, Centini G, Fernandes R, Murtada R, Zupi E, Akladios C (2016). Segmental and Discoid Resection are Preferential to Bowel Shaving for Medium-Term Symptomatic Relief in Patients With Bowel Endometriosis. J Minim Invasive Gynecol.

[5] Daraï E, Cohen J, Ballester M (2017). Colorectal endometriosis and fertility. Eur J Obs Gynecol Reprod Biol.

[6] Daraï E, Lesieur B, Dubernard G, Rouzier R, Bazot M, Ballester M (2011). Fertility after colorectal resection for endometriosis Results of a prospective study comparing laparoscopy with open surgery. Fertil Steril.

[7] Darwish B, Roman H (2016). Surgical treatment of deep infiltrating rectal endometriosis in favor of less aggressive surgery. Am J Obs Gynecol.

[8] De Cicco C, Corona R, Schonman R, Mailova K, Ussia A, Koninckx PR (2011). Bowel resection for deep endometriosis A systematic review. BJOG.

[9] Donnez J, Squifflet J (2010). Complications, pregnancy and recurrence in a prospective series of 500 patients operated on by the shaving technique for deep rectovaginal endometriotic nodules. Hum Reprod.

[10] Fourquet J, Báez L, Figueroa M, Iriarte I, Flores I (2011). Quantification of the impact of endometriosis symptoms on health-related quality of life and work productivity. Fertil Steril.

[11] Iversen ML, Seyer-Hansen M, Forman A (2017). Does surgery for deep infiltrating bowel endometriosis improve fertility A systematic review. Acta Obs Gynecol Scand.

[12] Jimenez-Gomez LM, Espin-Basany E, Trenti L, Martí-Gallostra M, Sánchez-García JL, Vallribera-Valls F Factors associated with low anterior resection syndrome after surgical treatment of rectal cancer. Color Dis.

[13] Kent A, Shakir F, Rockall T, Haines P, Pearson C, Rae-Mitchell W (2015). Laparoscopic Surgery for Severe Rectovaginal Endometriosis Compromising the Bowel A Prospective Cohort Study. J Minim Invasive Gynecol.

[14] Kondo W, Ribeiro R, Trippia C, Zomer MT (2012). Endometriose profunda infiltrativa distribuição anatômica e tratamento cirúrgico. Rev bras ginecol Obs.

[15] Koninckx PR, Martin DC (1992). Deep endometriosis a consequence of infiltration or retraction or possibly adenomyosis externa?. Fertil Steril.

[16] Kössi J, Setälä M, Mäkinen J, Härkki P, Luostarinen M (2013). Quality of life and sexual function 1 year after laparoscopic rectosigmoid resection for endometriosis. Color Dis.

[17] Lasmar RB, Lasmar BP, Keller Celeste R, Larbig A, De Wilde RL (2015). Validation of a score to guide endometriosis therapy for the non-specialized gynecologist. Int J Gynaecol Obs.

[18] Malzoni M, Di Giovanni A, Exacoustos C, Lannino G, Capece R, Perone C (2016). Feasibility and Safety of Laparoscopic-Assisted Bowel Segmental Resection for Deep Infiltrating Endometriosis A Retrospective Cohort Study With Description of Technique. J Minim Invasive Gynecol.

[19] Mangler M, Herbstleb J, Mechsner S, Bartley J, Schneider A, Köhler C (2014). Long-term follow-up and recurrence rate after mesorectum-sparing bowel resection among women with rectovaginal endometrios Int J Gynaecol. Obs.

[20] Meuleman C, Tomassetti C, Wolthuis A, Cleynenbreugel Van B, Laenen A, Penninckx F (2014). Clinical Outcome After Radical Excision of Moderate-Severe Endometriosis With or Without Bowel Resection and Reanastomosis. Ann Surg.

[21] Nirgianakis K, McKinnon B, Imboden S, Knabben L, Gloor B, Mueller MD (2014). Laparoscopic management of bowel endometriosis Resection margins as a predictor of recurrence. Acta Obs Gynecol Scand.

[22] Ret Dávalos ML, De Cicco C, D'Hoore A, De Decker B, Koninckx PR (2007). Outcome after rectum or sigmoid resection A review for gynecologists. J Minim Invasive Gynecol.

[23] Ribeiro PAA, Sekula VG, Abdala-Ribeiro HS, Rodrigues FC, Aoki T, Aldrighi JM (2014). Impact of laparoscopic colorectal segment resection on quality of life in women with deep endometriosis one year follow-up. Qual Life Res.

[24] Roman H, Loisel C, Resch B, Tuech JJ, Hochain P, Leroi AM (2010). Delayed functional outcomes associated with surgical management of deep rectovaginal endometriosis with rectal involvement Giving patients an informed choice. Hum Reprod.

[25] Roman H, Vassilieff M, Tuech JJ, Huet E, Savoye G, Marpeau L (2013). Postoperative digestive function after radical versus conservative surgical philosophy for deep endometriosis infiltrating the rectum. Fertil Steril.

[26] Silveira da Cunha Araújo R, Abdalla Ayroza Ribeiro HS, Sekula VG, da Costa Porto BT, Ayroza Galvão Ribeiro PA (2014). Long-Term Outcomes on Quality of Life in Women Submitted toLaparoscopic Treatment for Bowel Endometriosis. J Minim Invasive Gynecol.

[27] Soto E, Catenacci M, Bedient C, Jelovsek JE, Falcone T (2016). Assessment of Long-Term Bowel Symptoms After Segmental Resection of Deeply Infiltrating Endometriosis A Matched Cohort Study. J Minim Invasive Gynecol.

[28] Stepniewska A, Pomini P, Bruni F, Mereu L, Ruffo G, Ceccaroni M (2009). Laparoscopic treatment of bowel endometriosis in infertile women. Hum Reprod.

[29] Tarjanne S, Heikinheimo O, Mentula M, Harkki P (2015). Complications and long-term follow-up on colorectal resections in the treatment of deep infiltrating endometriosis extending to bowel wall. Acta Obs Gynecol Scand.

[30] Wu CY, Chang WP, Chang Y-H, Li C-P, Chuang CM (2015). The risk of irritable bowel syndrome in patients with endometriosis during a 5-year follow-up a nationwide population-based cohort study. Int J Color Dis.

